# Visual recognition of words learned with gestures induces motor resonance in the forearm muscles

**DOI:** 10.1038/s41598-021-96792-9

**Published:** 2021-08-26

**Authors:** Claudia Repetto, Brian Mathias, Otto Weichselbaum, Manuela Macedonia

**Affiliations:** 1 Department of Psychology, Università Cattolica del Sacro Cuore, Milan, Italy; 2 Chair of Cognitive and Clinical Neuroscience, Faculty of Psychology, Technical University Dresden, Dresden, Germany; 3 Research Group Neural Mechanisms of Human Communication, Max Planck Institute for Human Cognitive and Brain Sciences, Leipzig, Germany; 4 Department of Information Engineering, Johannes Kepler University, Linz, Austria; 5 Research Group Ilse Meitner Cognition and Plasticity, Max Planck Institute for Human Cognitive and Brain Sciences, Leipzig, Germany; 6 Linz Center of Mechatronics, Johannes Kepler University, Linz, Austria

**Keywords:** Cognitive neuroscience, Sensorimotor processing, Human behaviour

## Abstract

According to theories of Embodied Cognition, memory for words is related to sensorimotor experiences collected during learning. At a neural level, words encoded with self-performed gestures are represented in distributed sensorimotor networks that resonate during word recognition. Here, we ask whether muscles involved in gesture execution also resonate during word recognition. Native German speakers encoded words by reading them (baseline condition) or by reading them in tandem with picture observation, gesture observation, or gesture observation and execution. Surface electromyogram (EMG) activity from both arms was recorded during the word recognition task and responses were detected using eye-tracking. The recognition of words encoded with self-performed gestures coincided with an increase in arm muscle EMG activity compared to the recognition of words learned under other conditions. This finding suggests that sensorimotor networks resonate into the periphery and provides new evidence for a strongly embodied view of recognition memory.

## Introduction

Traditional perspectives in cognitive science describe human behaviour as mediated by cognitive representations^1^. Such representations have been defined as mental structures that encode, store and process information arising from sensory-motor systems^2^. According to these perspectives, information provided to perceptual systems about the environment is incomplete. As a result, the brain has the essential role of transforming this information into cognitive representations, which enable rapid and accurate behaviours. In recent years, embodied approaches have claimed that perception, action and the environment jointly contribute to cognitive processes^3,4^, highlighting a change in our understanding of the role of the body in cognition. Rather than serving as an unreliable source of information—as in traditional cognitive theories^1,5,6^—the body has become a substantial contributor to the construction of knowledge^7^.

One of the core cognitive functions contributing to the construction of knowledge is memory. According to embodied theories^8,9^, memory processes are modal, i.e., the content of a particular memory is closely related to the sensorimotor experiences that have been collected in order to generate the memory^10^. Laboratory research has provided evidence that the body plays a key role in memory processes^11–13^. For example, studies investigating effects of adding a self-performed action or a gesture to a linguistic stimulus during encoding have revealed a phenomenon known as the enactment effect^14^. Indeed, studies on memory for action conducted in the early 1980s demonstrated that associating a self-performed action (self-performed task) with a verbal stimulus during encoding has a beneficial effect on word retention, compared to either observing someone else performing the action (experimenter-performed task), or to verbal-only encoding^11,14,15^. Thus, learners tend to remember the sentence “grasp the apple” better if they perform the action of grasping an (imaginary) apple than if they see an experimenter performing the same action or if they only read/hear the sentence. The enactment effect is robust and it has been documented in a variety of memory tasks, including free recall^16–20^, cued recall^21^, and word recognition^22–27^, and in a variety of populations, young and elderly individuals^28^, patients with Korsakoff syndrome^29^, and Alzheimer’s patients^30^.

The pairing of actions with verbal information is not only beneficial for memory. The performance of gestures^31^ during the learning of novel native^32,33^ and second language (L2) word also supports vocabulary acquisition (see for a review^34^). Macedonia and Klimesch^35^ trained college students to memorize 36 novel words in “Tessetisch,” an artificial vocabulary corpus created in order to avoid associations with natural languages. The words were encoded under two different learning conditions: audiovisual encoding and audiovisual encoding paired with a self-performed gesture. The words belonged to several different vocabulary classes including concrete nouns, verbs, adjectives and prepositions. Whereas gestures related to concrete nouns and verbs were considered iconic, gestures associated with adjectives and prepositions were considered symbolic. Additionally, while the iconic gestures were hand movements representing attributes of the referent, such as shape or function^36,37^, the symbolic gestures represented the meaning based on cultural agreements or habits^38^. Memory tests administered at five time points distributed over 14 months demonstrated that gestures significantly enhanced vocabulary learning in both the short- and long-term. Similar results were reported by Repetto and collaborators^39^, who investigated the effect of gestures on the learning of abstract words in a second language. After a one-hour training period, performance on a recognition task was shown to benefit from enactment-based word encoding.

The enactment effect was first explained by the Motor Trace theory^40^ proposed by Engelkamp. According to this theory, verbal stimuli can be encoded along several routes: visual, semantic and motor. Following the account proposed by Paivio—the Dual Coding Theory—two modalities are more efficient than one^41^. Engelkamp and Zimmer reasoned that three modalities (visual, semantic, and motor) are even more efficient than two^42,43^. Motor encoding was proposed to generate a motor trace when the subject performs an action. This trace becomes a part of a word’s representation. In other words, when enacting a word, movement kinematics, in the form of kinaesthetic feedback, help to establish a multimodal representation including a motor trace. The multimodal representation subsequently helps to improve item-specific processing, leading to enhanced memory performance.

The Motor Trace theory bears similarity to the Reactivation theory of sensorimotor learning^44^. The latter postulates that brain areas active during encoding of words are later re-activated when the items are recalled^44,45^. This is to say that, if an item has been associated with specific sensorimotor experiences during encoding, the same sensory and motor brain regions connected to the learning experience will become active during recall. Thus, in the case of the enactment effect, motor regions that support gesture execution during word encoding would be expected to be reactivated when a word is retrieved from memory. This effect has been shown in brain imaging studies^46–48^. Using positron emission tomography (PET) imaging, Nilsson and colleagues^46^ found that motor brain areas including the primary motor cortices responded during the retrieval of items that had been encoded through enactment. Similar findings were reported by Masumoto and colleagues with magnetencephalography (MEG)^47^. Evidence for the role of the motor cortices in the representation of enacted words also comes from studies on word learning in L2. In an fMRI study, Macedonia and colleagues trained participants on L2 words in association with iconic gestures. Participants showed heightened responses within the premotor cortices during L2 word recognition after learning^48^. Other studies have reported that the perception of previously enacted items elicits responses within the inferior parietal cortex, supramarginal gyrus^49^, subcortical regions, basal ganglia, and cerebellum^50^.

The reactivation of motor regions during the retrieval of enacted items is one example of a more general phenomenon termed *motor resonance* (MR). This term was coined in parallel with the discovery of mirror neurons^51,52^, i.e., neurons that become active both when individuals performs an action themselves and when they observe another individual performing the same action^53,54^. The visuomotor transformation at the base of this effect has been referred to as motor simulation or resonance^55^. According to Rizzolatti^56^, MR is the key to successful social interaction. The concept of MR has been widely investigated, with the purpose of unravelling the conditions under which it emerges, and the factors that may influence its occurrence^57^. Current evidence suggests that MR is not an all-or-nothing phenomenon. For example, MR is modulated by an observer’s judgments about the consequences of the action^58^ and it is greater for observed actions that match a specific observer’s motor expertise, compared to actions that do not belong to the observers’ motor expertise^59^; furthermore, MR is elicited by static pictures that depict actions^60^.

MR also appears to be linked to the specific meaning of actions rather than to the observation of a motor pattern per se. Support for this aspect of MR comes from studies demonstrating enhanced activity in the motor cortices in response to stimuli that refer to actions without overtly displaying them. Haueisen and Knösche^61^ compared the cortical activity of pianists and non-pianists listening to piano compositions. The pianists exhibited enhanced activity in the hand area of the primary motor cortex while listening if they had already practiced the music. Moreover, brain surface current density reconstructions revealed a somatotopic dissociation: listening to tones typically produced by the thumb resulted in increased activity in the thumb area of the motor cortex, while listening to tones typically produced by the little finger resulted in increased activity in the area known to control that finger. These findings indicate that simply perceiving a stimulus such as music that has been stored in memory together with a motor pattern spontaneously triggers the reactivation of the same motor pattern.

The role of MR in semantic processing of language is debated, especially in the case of abstract words and concepts, which lack intrinsic sensorimotor associations^62,63^. However, MR is known to occur during the perception of action semantics in language. Brain imaging experiments have found increased activity in the motor regions elicited by words describing action execution^64–68^. This is to say that action-related semantics in language can induce a somatotopically fine-grained MR^69,70^. The localization of brain activity associated with MR elicited by language is not fully consistent across studies. Some authors have reported enhanced activity within the premotor cortex^71,72^, which could endow individuals with information needed to prepare upcoming actions. Other studies have identified more extensive activity involving also the primary motor cortex^61,65,66,68,73^, suggesting that MR can extend further towards the nervous system’s motor pathways and closer to the musculature responsible for movement execution.

To our knowledge, only one study^74^ has investigated whether motor responses during semantic processing extend to the muscles, thus involving also the peripheral motor system. In a study conducted by Stins and Beek^74^, participants lay supine and read arm- and leg-related verbs; the task was either a semantic judgment (i.e., “say yes if the verb describes an action performed with the arm/leg”) or a phonological judgment (i.e., “say yes if the verb contains the letter R”). Surface EMG signals were recorded from the participants’ upper and lower limb muscles. The authors expected to find increased spontaneous motor responses in arm and leg muscles, reflected in enhanced EMG activity during the semantic processing of arm- and leg-related action verbs, respectively. Contrary to their predictions, they found that hand-related and leg-related words elicited a small decrease in the corresponding muscle activity during semantic judgments compared to phonological judgments. This finding was interpreted as suggesting that motor inhibition occurred while reading action verbs, similar to what has been reported in studies on motor imagery^75^. The authors concluded that even if semantic and motor processes share common neural substrates, the body periphery is likely to be insulated from motor activity occurring during perception.

The present study aimed to show the effects of enactment in the body periphery after sensorimotor word learning. We tested whether written words that were previously encoded through enactment, by the performance of iconic gestures, would elicit increased peripheral motor activity. In line with previous studies on L2 learning following gesture execution^76^ and gesture observation^39^, we included not only concrete words but also abstract words. They challenge MR theories as they are not intrinsically linked to sensorimotor experiences. In our study, participants were instructed to remember the words in their native language. Each word was encoded by reading its written word form while viewing a related image (VI condition), observing a related gesture (VGO condition), or observing and performing a related gesture (VGOP condition). In a control condition (V), participants read the written words in the absence of any complementary stimulus. Surface muscle EMG signals were recorded from both arms during a word recognition task administered after encoding.

We had two primary predictions and one secondary prediction. First, we expected increased peripheral motor activity while participants recognized words that they previously encoded through self-performed gestures. Second, based on previous findings reporting positive effect of gestures on both concrete and abstract word memorisation^35,39^, we predicted that an increase in peripheral motor activity would occur for both concrete and abstract words. Such as a result would suggest that MR is not limited to the brain, but rather spreads to the body’s periphery. Our secondary prediction was that, in line with previous studies^34^, words encoded in association with self-performed gestures would be better memorized than words encoded in the other conditions.

## Results

**Figure 1:**
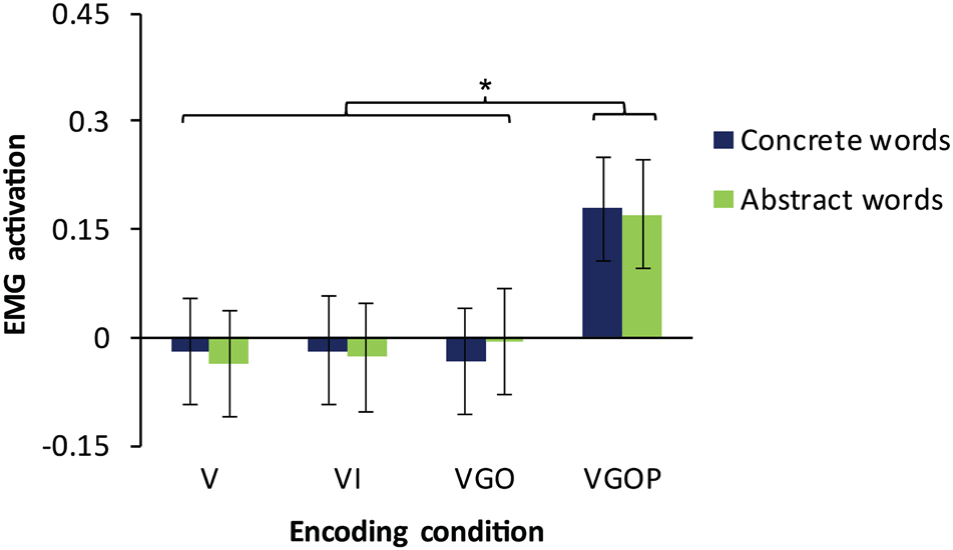
Effects of encoding condition and word type on EMG responses. Performing and observing gestures during word encoding (VGOP) enhanced EMG responses at test compared with encoding based only on the written wordform (V), encoding enriched with illustrations (VI), and encoding enriched with observed gestures (VGO). *n* = 28 participants. Error bars represent one standard error of the mean. *p < .05.

We first assessed effects of encoding condition and word type on EMG responses during the post-encoding recognition task. Mean EMG responses by encoding and word type conditions are shown in Fig. 1. Estimated fixed and random effects of the mixed effects modelling on mean EMG responses are shown in Table 1. The mixed model revealed an effect of encoding driven by the VGOP condition, *β* = 0.21, *t* = 2.03, 95% CI [0.01 0.41]. The Wald *χ*^2^ test yielded a significant main effect of encoding, *χ*^2^ (3, *N* = 28) = 11.58, *p* = 0.009. Tukey post-hoc tests indicated that EMG responses were significantly enhanced following VGOP encoding compared to V encoding, *t* = 2.84, *p* = 0.02, VI encoding, *t* = 2.73, *p* = 0.03, and VGO encoding, *t* = 2.71, *p* = 0.03. VGO encoding did not significantly enhance EMG responses compared with V encoding, *t* = 0.14, *p* = 0.99, or VI encoding, *t* = 0.06, *p* = 0.99. The mixed effects model of EMG responses did not reveal any significant influences of the word type factor or interactions between encoding and word type factors. A Wald *χ*^2^ test for an effect of word type was not significant, *χ*^2^ (1, *N* = 28) = 0.001, *p* = 0.98, and a test for an encoding condition × word type interaction was also not significant, *χ*^2^ (3, *N* = 28) = 0.13, *p* = 0.99.

**Table 1:** Linear mixed effects regression model testing the effects of encoding condition and word type on EMG responses during the visual vocabulary recognition task.

Fixed effects					Random effects			
	Estimate	SE	*t*	CI			Variance	SD
Intercept	.026	.040	.64	− .05, .11	Participant	Intercept	.008	.092
Encoding [VI]	.010	.102	.10	− .19, .21	Stimulus	Intercept	< .001	< .001
Encoding [VGO]	.033	.101	.33	− .16, .23	Residual		.801	.895
Encoding [VGOP]	.208	.103	2.03*	.01, .41				
Word type	.001	.050	.03	− .10, .10				
Encoding [VI]:Word type	− .008	.143	− .06	− .29, .27				
Encoding [VGO]:Word type	− .046	.141	− .33	− .32, .23				
Encoding [VGOP]:Word type	− .093	.143	− .07	− .29, .27				

V, visual; VI, image; VGO, gesture observation; VGOP, gesture observation and self-performance. *n* = 28 participants. *p < .05.

**Figure 2:**
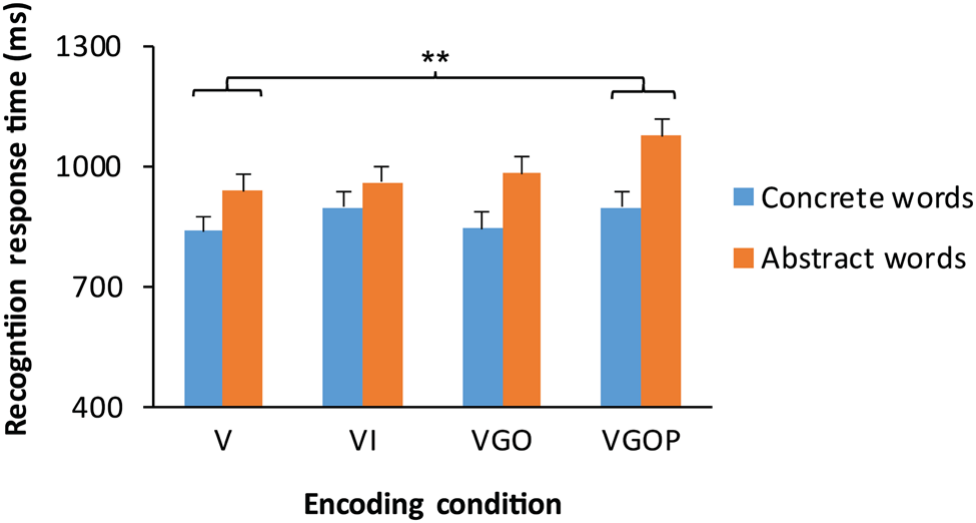
Effects of encoding condition and word type on recognition response time. Mean response time (ms) for target words in the recognition task by encoding condition and word type. Words encoded by performing and observing gestures (VGOP) were recognized significantly more slowly than words learned only visually (V). *n* = 28 participants. Error bars represent one standard error of the mean. **p < .01.

We next tested whether encoding condition and word type factors modulated recognition response times. Estimated fixed and random effects of the mixed effects modelling of recognition response times are shown in Table 2. The model revealed an effect of encoding driven by the VGOP condition, *β* = 137.2, *t* = 3.18, 95% CI [53 222]. The Wald *χ*^2^ test yielded a significant main effect of encoding, *χ*^2^ (3, *N* = 28) = 11.53, *p* = 0.009. Tukey post-hoc tests indicated that participants recognized VGOP-encoded words significantly more slowly than V-encoded words, *t* = -3.47, *p* = 0.003, shown in Fig. 2. The Wald *χ*^2^ test yielded a significant main effect of word type, *χ*^2^ (1, *N* = 28) = 16.04, *p* < 0.001, indicating that participants recognized concrete words significantly faster than abstract words. Encoding condition and word type factors did not significantly interact, *χ*^2^ (3, *N* = 28) = 4.64, *p* = 0.20.

**Table 2:** Linear mixed effects regression model testing the effects of encoding condition and word type on response time in the recognition task.

Fixed effects					Random effects			
	Estimate	SE	*t*	CI			Variance	SD
Intercept	988.3	32.6	30.35	924, 1054	Participant	Intercept	16,887	130
Encodingt[VI]	22.1	41.9	.53	− 60, 104	Stimulus	Intercept	7134	84
Encoding [VGO]	44.2	41.8	1.06	− 38, 126				
Encoding [VGOP]	137.2	43.2	3.18**	53, 222				
Word type	− 118.6	29.4	− 4.04***	− 177, − 60				
Encoding [VI]:Word type	36.2	55.3	.65	− 72, 145				
Encoding [VGO]:Word type	− 36.2	55.3	− .65	− 145, 72				
Encoding [VGOP]:Word type	− 78.6	56.3	− 1.40	− 189, 32				

V, visual; VI, image; VGO, gesture observation; VGOP, gesture observation and self-performance. *n* = 28 participants. **p < .01; *** *p* < .001.

**Figure 3:**
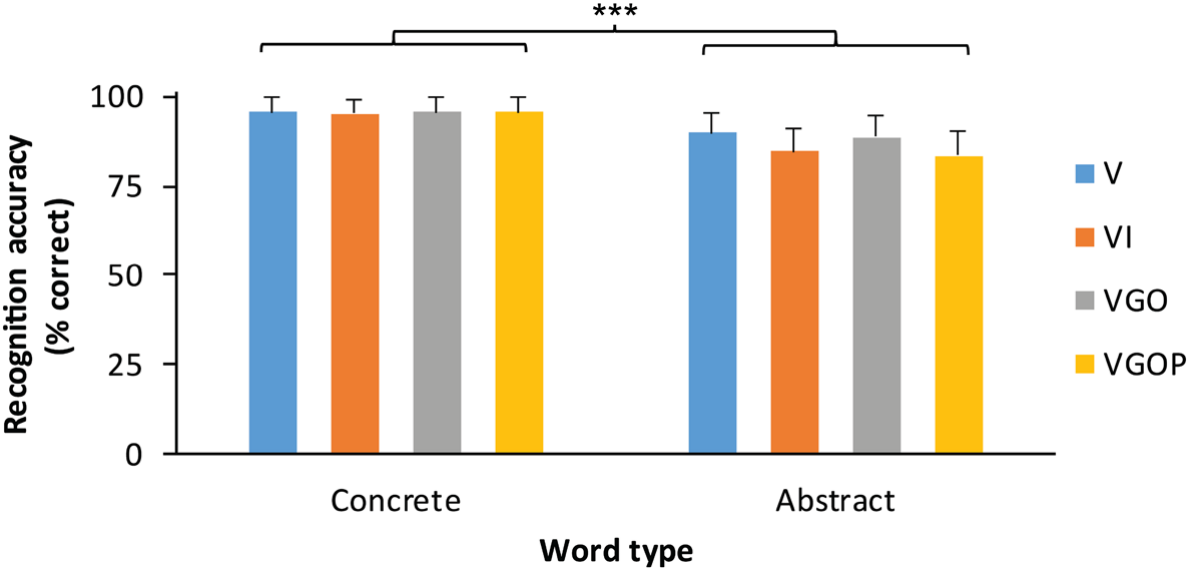
Effects of learning enrichment and word type on recognition accuracy in the non-EMG recognition task. Words correctly recognized (% correct) per condition in the recognition task by enrichment condition and word type. Participants recognized significantly more concrete words than abstract words. Encoding conditions: V = visual, VI = image, VGO = gesture observation, VGOP = gesture observation and self-performance. *n* = 28 participants. Error bars represent one standard error of the mean. ***p < .001.

We also tested whether encoding condition modulated accuracy in the memory recognition task. Estimated fixed and random effects of the mixed effects modelling of accuracy in the non-EMG recognition task are shown in Table 3. A Wald *χ*^2^ test indicated no significant effect of encoding condition, *χ*^2^ (3, *N* = 28) = 6.60, *p* = 0.09. There was, however, a significant effect of word type, *χ*^2^ (1, *N* = 28) = 53.70, *p* < 0.001, shown in Fig. 3. Participants recognized concrete words significantly more accurately than abstract words. Encoding condition and word type factors did not significantly interact, *χ*^2^ (3, *N* = 28) = 2.68, *p* = 0.44.

**Table 3:** Linear mixed effects regression model testing the effect of encoding condition and word type on accuracy in the non-EMG recognition task.

Fixed effects					Random effects			
	Estimate	SE	*z*	CI			Variance	SD
Intercept	2.05	.22	9.16	1.61, 2.48	Participant	Intercept	1.06	1.03
Enrichment [VI]	− .53	.30	− 1.79	− 1.11, .05	Stimulus	Intercept	.29	.54
Enrichment [VGO]	− .50	.31	− 1.61	− 1.11, .11				
Enrichment [VGOP]	− .61	.29	− 1.91	− 1.21, .02				
Word type	1.40	.19	7.28***	1.02, 1.78				
Enrichment [VI]:Word type	.43	.54	.79	− .63, 1.48				
Enrichment [VGO]:Word type	.50	.55	.90	− .58, 1.58				
Enrichment [VGOP]:Word type	.89	.54	1.63	− .18, 1.95				

V, visual; VI, image; VGO, gesture observation; VGOP, gesture observation and self-performance. *n* = 28 participants. ***p < .001.

**Figure 4:**
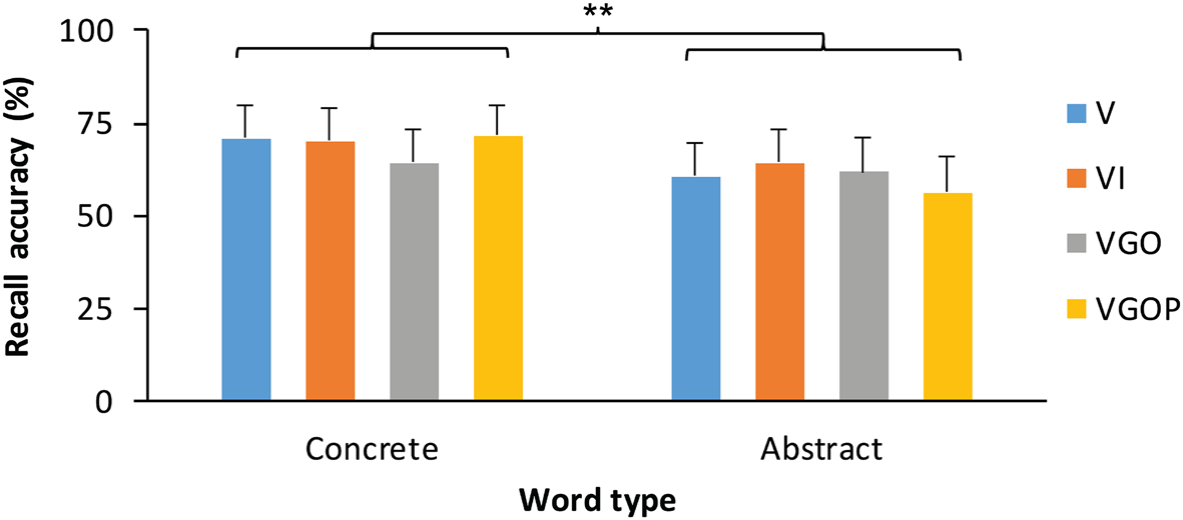
Effects of encoding condition and word type on free recall accuracy. Words correctly recalled (%) per condition in the free recall task by encoding condition and word type. Participants recalled significantly more concrete words than abstract words. Each of the encoding conditions included 16 words. Assignment of words to encoding conditions was counterbalanced across participants. Encoding conditions: V = visual, VI = image, VGO = gesture observation, VGOP = gesture observation and self-performance. *n* = 28 participants. Error bars represent one standard error of the mean. **p < .01.

We next tested whether encoding condition and word type factors modulated free recall performance. Estimated fixed and random effects of the mixed effects modelling of free recall response accuracy are shown in Table 4. A Wald *χ*^2^ test indicated no significant main effect of encoding type, *χ*^2^ (3, *N* = 28) = 1.68, *p* = 0.64, shown in Fig. 4. The Wald *χ*^2^ test yielded a significant main effect of word type, *χ*^2^ (1, *N* = 28) = 10.23, *p* = 0.001, indicating that participants recalled significantly more concrete words than abstract words. Encoding condition and word type factors did not significantly interact, *χ*^2^ (3, *N* = 28) = 4.97, *p* = 0.17.

**Table 4:** Linear mixed effects regression model testing the effect of encoding condition and word type on accuracy in the free recall task. V = visual, VI = image, VGO = gesture observation, VGOP = gesture observation and self-performance. *n* = 28 participants. **p < .01.

Fixed effects					Random effects			
	Estimate	*SE*	*z*	CI			Variance	*SD*
Intercept	.58	.20	2.88	.19, .98	Participant	Intercept	.83	.91
Encoding [VI]	.14	.31	.45	− .47, .76	Stimulus	Intercept	.16	.40
Encoding [VGO]	.03	.31	.10	− .57, .64				
Encoding [VGOP]	− .29	.27	− 1.08	− .83, .24				
Word type	.45	.15	3.03**	.16, .74				
Encoding [VI]:Word type	− .26	.33	− .79	− .92, .39				
Encoding [VGO]:Word type	− .46	.33	− 1.41	− 1.10, .18				
Encoding [VGOP]:Word type	.19	.32	.59	− .44, .82

Finally, we tested for correlations between participants’ mean EMG responses and their accuracy and response times in the non-EMG recognition task by encoding condition. There were no significant correlations of EMG responses with accuracy or response times in the non-EMG recognition task (all *p*’s > 0.13, Bonferroni corrected). Participants’ mean EMG responses also did not significantly correlate with their accuracy in the free recall task (all *p*’s > 0.09, Bonferroni corrected).

## Discussion

The current study investigated MR in the peripheral motor system using EMG in the context of native language word encoding and retrieval. German-speaking participants encoded concrete and abstract German words under several conditions. In a control condition, participants saw only written words (V), and in other conditions, the written word was presented along with an image illustrating the word’s meaning (VI) or a gesture representing the word’s meaning, which was either observed only (VGO) or performed and observed (VGOP). Word retention was tested with free recall and visual word recognition tasks. Participants’ forearm muscle EMG activity was then recorded as they completed a visual word recognition task. The study yielded two main findings. First, words encoded with self-performed gestures elicited greater muscle activation than words encoded in all other training conditions. Second, interestingly, participants recognized words encoded with self-performed actions more slowly than words encoded by only viewing the written word forms, and accuracy in both the recognition and free recall tasks did not differ across encoding conditions. Each of these findings are discussed in light of our predictions.

The results confirm our primary prediction: increased peripheral motor activity coincided with the recognition of words encoded through self-performed gestures. This suggests that MR spreads to the body’s periphery and is consistent with theories of embodied cognition. They propose that our bodily experiences greatly contribute to cognitive processes^77,78^. This finding is also consistent with previous neuroimaging studies that report enhanced activity within the motor cortices during the recall and recognition of recently-enacted words^46–48^.

In our view, increased peripheral motor activity during the recognition of words learned through enactment indicates that both the brain and the effectors participate in memory recognition processes. During encoding, participants associated a self-performed gesture with a word. This presumably resulted in a motor memory trace^12^. The primary motor cortex (M1) likely activated the muscles needed to perform that gesture. During word retrieval, brain regions involved in encoding, namely motor areas, were reactivated. This, in turn, resulted in a sub-threshold level of muscle activity. This effect occurred only when the participants performed a gesture during encoding, and not when they had merely observed an actress performing the related gesture. In the latter case, since the gesture was not executed during the learning phase, the pattern of motor commands needed to perform the gesture by the subject was not associated to the word and did not result in muscle reactivation. This interpretation fits with embodied accounts of human cognition such as the Perceptual Symbol System account proposed by Barsalou^10^, which focuses on the role of simulation in cognition. Simulation is defined as the process allowing the re-enactment of previously acquired perceptual, motor and introspective bodily states. This interpretation is also consistent with reactivation theories of multisensory learning^44^. They postulate an overlap between brain areas activated during encoding and those activated during retrieval of the same items.

Our results extend those reported by Stins and Beek^74^. In that study, the authors showed that encoding conditions play a critical role in the emergence of MR. Stins and Beek observed a reduction of EMG activity during semantic processing of action verbs congruent with particular effectors. Our results depart from these findings. In fact, the recognition task used in the present study and the decision task used by Stins and Beek differ in several key ways. In the study by Stins and Beek, EMG signals were recorded while participants read and made semantic judgments about words whose meanings were related to arm or foot actions. Thus, the absence of peripheral motor activation would indicate that muscles are not part of the multisensory network that contributes to word semantics^79^, or that the reading of action verbs acts invokes motor imagery processes, thereby inducing motor suppression^75^. In our experiment, participants first encoded words and then performed a recognition task during which EMG signals were recorded. EMG activity was therefore not related to the semantic content of words themselves, but rather related to the learning experience. In other words, the peripheral motor activity in the current study was due to the re-instantiation of the sensory-motor experiences occurred during encoding. Support for this interpretation comes from the results: both concrete and abstract words encoded through self-performed gestures—and not only those words possessing intrinsic motor associations—triggered spontaneous muscle activity. Thus, enactment may create motor traces that spread to the periphery, whereas the processing of word semantics is confined to brain networks established during language acquisition. In the Stins and Beek’s study^71^, during the experiment, participants lay in a supine position. This position is incompatible with the execution of the action verbs used in the study. Furthermore, the instruction to lay may have suppressed spontaneous motor activation. Instead, in our protocol, the participants’ posture during the EMG task was compatible with the body position used to execute the gestures during training. Other literature confirms the importance of the body position during word processing. For example, Lachmair and collaborators^80^ prompted participants to recall literal and metaphorical concepts related to the up-down vertical dimension in two different body positions, either upright or head-down tilted. Memory performance evidenced a double dissociation between word meaning and body position: the UP-words were more likely to be recalled in the upright position, and the down-words were more likely to be recalled in the tilted position. The authors interpreted this result as depending on the congruence between the bodily experiences and the meanings of the spatial words. Future studies will investigate the role of this factor in semantic judgements by manipulating the body position in which words are encoded and recalled.

Our second prediction was also confirmed: increases in peripheral motor activity in our study were related to encoding experience rather than word category, i.e. whether a word was concrete or abstract. This was expected on the base of previous studies that reported impact of gestures on both abstract and concrete words memorisation^35,39^. This finding supports the view that enactment during encoding involves the peripheral motor system also during subsequent recognition, regardless of the class of an encoded word.

This result is consistent with the Theory of Integrated Communication Systems by McNeill^81^ and later by Kita^82^. The theory postulates that both spoken language comprehension and the production of gestures are managed by the same unique control system. Therefore, gestures are expected to facilitate language comprehension and production, as well as language learning. Paivio’s Dual Coding Theory^83^ suggests that concrete words are better memorized because of the existence of a dual code, i.e. a linguistic and a sensorimotor imagery code. Whereas concrete words would activate both the linguistic and sensorimotor codes, abstract words would only activate the linguistic code. The slower response times for abstract words in the VGOP condition are potentially explained by this aspect of Paivio’s theory.

In the Language Situated Simulation (LASS) hypothesis proposed by Barsalou et al.^10^ and in the Words As Tools (WAT) hypothesis by Borghi and Cimatti^84^, concrete and abstract words are assumed to possess differing cognitive representations. These characteristics lead to differences in word learning and usage. Concrete words have direct relations with the objects or actions they refer to, whereas abstract words are only indirectly related to their referents. However, abstract words can become more like concrete words if they are disambiguated by inserting them into a more concrete context, i.e., in a context in which gestures are performed. Gestures essentially make the words’ meanings more graspable and narrower^85–89^. Thus, memorizing abstract words by performing gestures might change their cognitive representations and make them more similar to concrete words. Interestingly, in a transcranial magnetic stimulation (TMS) study, De Marco and colleagues^90^ investigated the excitability of the motor cortex during abstract word comprehension. They expected a modulation of motor cortex excitability if a word had previously been connected to a semantically-related gesture. Gestural motor representations were found to facilitate performance in a lexical decision task (please see Mathias et al^91^ for similar effects of TMS on the learning of both abstract and concrete L2 words). These findings further support the view that motor features can become a critical part of abstract word representations if the words become associated with self-performed gestures during learning.

Our secondary prediction was not supported by the current findings. The amount of time participants took to recognize words that had been encoded using enactment was longer than the time they took to recognize words encoded based only on visual wordforms. Longer response times in the recognition task for words encoded using enactment may be due to interference of the arm muscle activity with the execution of actions needed to give the response. Such interference effects have previously been observed in the context of language perception^92^. For example, some studies have demonstrated that processing action words interferes with the execution of a concomitant action performed with the same effector. In fact, hand action words interfere with the execution of hand movements, and foot action words interfere with the execution of foot movements^93–97^. These studies conclude that when multiple motor programs compete, i.e., one activated as part of the semantic word network and one activated to provide a response, the switch from the first to the second slows response time. Another recent study observed a similar interference effect during a semantic judgment task: visual interference (the presentation of an array of meaningless shapes) slowed down reaction times for words as a function of the degree of the visual experience individuals typically have collected with the words’ referents^98^. Our results extend this finding to suggest that motor interference can occur also in word memory tasks.

In addition, increases in peripheral motor activity in the current study did not correspond to differences in memory performance across encoding conditions. We speculate that the lack of an effect of gestural encoding on memory accuracy in the present study may stem from the fact that accuracy in the memory recognition task was at ceiling across conditions. This was due to the low number of stimuli tested. A previous study that made use of a larger number of stimuli (30 words per condition) revealed memory enhancement for enacted words compared to non-enacted words^99^. This could potentially also be due to the significantly shorter training in the current study compared to previous studies, in which training lasted up to 5 days^91,99^ or longer^35^. Future research should test effects of longer or shorter training periods, as well as larger sets of vocabulary in both native and foreign languages, to further probe the relationship between memory performance and muscle activity. Additionally, as gestures in the current study were performed with the hands, arms and shoulders, future research could investigate enactment that makes use of alternate effectors such as the legs and feet.

## Conclusion

This is the first study to directly investigate the activation of peripheral motor system during the recognition of words encoded using enactment. The findings show that MR, which so far has been confined to the cortical motor system, may under some circumstances also recruit specific effectors. In conclusion, this study provides new evidence that memories are grounded in the body, in support of embodied cognition theories. Memory for enacted words may depend not only on the mind, but also on the body.

## Methods

### Participants

An a priori power analysis was conducted (http://jakewestfall.org/two_factor_power) based on a design in which random items were nested within condition and random participants crossed between conditions (CNC design)^100^. The analysis indicated that a minimum sample of 28 participants was needed to detect a medium-sized effect (*d* = 0.6) with a general linear mixed model including 64 random items.

Thirty participants were recruited from the Johannes Kepler University of Linz community. Two participants were excluded from the analyses due to the cessation of eye-tracking data recording shortly after the start of the experiment, leaving a total of 28 participants included in all analyses. The 28 participants (*M* age = 23.27 years, *SD* = 2.81 years, 14 females) were all native German speakers. All of the participants reported normal or corrected-to-normal vision. None reported any history of neurological or psychiatric diseases. The participants were naïve with respect to the aims and hypotheses of the study.

The experimental protocol was approved by the Internal Committee of the Department of Information Engineering of the Johannes Kepler University (JKU) of Linz. At the time of data collection (2017), the JKU was a technical university with no medicine faculty. Therefore, there was no Ethic Committee. Behavioural experiments with healthy subjects were presented and approved within the Departments conducting them. The Internal Committee of the Department of Information Engineering at the Johannes Kepler University licensed studies with healthy subjects (no patients) that posed neither physical nor psychic risks for the participants. Physical risks include harmful interaction within the experiment that might lead to physical harm. Psychic risks include experimental interactions that might provoke any psychic trauma or any temporary or permanent damage to the psyche of the subjects. The present learning experiment was conducted with participants sitting in front of a screen and memorizing words. This behaviour was considered as not leading to any physical or psychic harm for the young adults taking part into the experiment. All participants provided written, informed consent prior to participation. The study was conducted in accordance with the Helsinki Declaration of 1975, as revised in 2008.

### Stimuli

Sixty-four German words, shown in Table 5, were selected for the experiment. Half of them were concrete words denoting manipulable and non-manipulable objects (objects that can be touched but not handled, i.e. a balcony); the other half were abstract words, denoting mental processes, symbolic activities, relations and values. The lengths of concrete and abstract German words were identical (concrete *M* = 2.62 syllables, *SD* = 0.71 syllables; abstract: *M* = 2.62 syllables, *SD* = 0.71 syllables). Frequencies of the concrete and abstract words in written German were similar (concrete frequency score: *M* = 11.1, *SD* = 1.23, range 9 to 14; abstract frequency score: *M* = 10.97, *SD* = 1.23, range: 9 to 15; http://wortschatz.uni-leipzig.de/de). Sixty-four additional filler words were included in the experiment. Filler words were 32 abstract and 32 concrete words, matched to the words trained for number of syllables and frequency score.

**Table 5:** The complete set of stimuli.

German word	Syllables	Frequency score	English translation	Stimulus type	Word type	Block
Anforderung	4	11	Requirement	Target	Abstract	1
Ankunft	2	11	Arrival	Target	Abstract	1
Aufwand	2	11	Expenditure	Target	Abstract	1
Aussicht	2	10	View	Target	Abstract	1
Befehl	2	12	Command	Target	Abstract	1
Bestimmung	3	12	Designation	Target	Abstract	1
Disziplin	3	11	Discipline	Target	Abstract	1
Empfehlung	3	11	Advice	Target	Abstract	1
Gleichgültigkeit	4	14	Indifference	Target	Abstract	2
Talent	2	11	Talent	Target	Abstract	2
Tendenz	2	11	Trend	Target	Abstract	2
Triumph	2	11	Triumph	Target	Abstract	2
Übung	2	11	Exercise	Target	Abstract	2
Methode	3	10	Method	Target	Abstract	2
Partnerschaft	3	10	Partnership	Target	Abstract	2
Therapie	3	10	Therapy	Target	Abstract	2
Aufmerksamkeit	4	10	Caution	Target	Abstract	3
Besitz	2	10	Ownership	Target	Abstract	3
Bitte	2	10	Favor	Target	Abstract	3
Geduld	2	11	Patience	Target	Abstract	3
Rücksicht	2	11	Respect, respect	Target	Abstract	3
Absage	3	10	Cancellation	Target	Abstract	3
Korrektur	3	12	Correction	Target	Abstract	3
Gedanke	3	11	Thought	Target	Abstract	3
Information	4	10	Information	Target	Abstract	4
Vorwand	2	12	Pretext	Target	Abstract	4
Warnung	2	12	Warning / caution	Target	Abstract	4
Wohlstand	2	11	Wellbeing	Target	Abstract	4
Wohltat	2	15	Beneficence	Target	Abstract	4
Tatsache	3	9	Fact	Target	Abstract	4
Teilnahme	3	9	Participation	Target	Abstract	4
Theorie	3	11	Theory	Target	Abstract	4
Eintrittskarte	4	14	Ticket	Target	Concrete	1
Ampel	2	12	Traffic light	Target	Concrete	1
Balkon	2	11	Balcony	Target	Concrete	1
Bildschirm	2	11	Screen	Target	Concrete	1
Decke	2	11	Blanket	Target	Concrete	1
Anhänger	3	10	Pendant	Target	Concrete	1
Briefkasten	3	13	Mailbox	Target	Concrete	1
Gemälde	3	11	Painting	Target	Concrete	1
Sonnenbrille	4	13	Sunglasses	Target	Concrete	2
Flasche	2	11	Bottle	Target	Concrete	2
Flugzeug	2	10	Plane	Target	Concrete	2
Geschenk	2	10	Gift	Target	Concrete	2
Kabel	2	11	Electric wire	Target	Concrete	2
Katalog	3	11	Catalog	Target	Concrete	2
Maschine	3	10	Machine	Target	Concrete	2
Papier	3	10	Paper	Target	Concrete	2
Fernbedienung	4	13	Remote control	Target	Concrete	3
Denkmal	2	11	Monument	Target	Concrete	3
Faden	2	12	Thread	Target	Concrete	3
Fahrrad	2	11	Bicycle	Target	Concrete	3
Fenster	2	9	Window	Target	Concrete	3
Gitarre	3	11	Guitar	Target	Concrete	3
Handtasche	3	13	Purse	Target	Concrete	3
Kamera	3	9	Camera	Target	Concrete	3
Tageszeitung	4	10	Daily newspaper	Target	Concrete	4
Kasse	2	10	Cashbox	Target	Concrete	4
Kleidung	2	10	Dress	Target	Concrete	4
Koffer	2	11	Suitcase	Target	Concrete	4
Maske	2	12	Mask	Target	Concrete	4
Schublade	3	12	Drawer	Target	Concrete	4
Straßenbahn	3	12	Tram	Target	Concrete	4
Telefon	3	10	Phone	Target	Concrete	4
Abenteuer	4	10	Adventure	Filler	Abstract	–
Anfrage	3	9	Inquiry	Filler	Abstract	–
Berufung	3	10	Vocation	Filler	Abstract	–
Bestätigung	4	11	Confirmation	Filler	Abstract	–
Charakter	3	10	Character	Filler	Abstract	–
Ehre	2	10	Honor	Filler	Abstract	–
Empfindung	3	16	Sensation	Filler	Abstract	–
Erfahrung	3	9	Experience	Filler	Abstract	–
Erleichterung	4	12	Relief	Filler	Abstract	–
Ersparnis	3	14	Savings	Filler	Abstract	–
Erwartung	3	12	Expectation	Filler	Abstract	–
Fantasie	3	12	Fantasy	Filler	Abstract	–
Gebet	2	11	Prayer	Filler	Abstract	–
Geheimnis	3	10	Secret	Filler	Abstract	–
Glaube	2	11	Faith	Filler	Abstract	–
Großzügigkeit	4	14	Generosity	Filler	Abstract	–
Güte	2	12	Quality	Filler	Abstract	–
Irrtum	2	12	Mistake	Filler	Abstract	–
Mangel	2	11	Defect	Filler	Abstract	–
Mitleid	2	12	Pity	Filler	Abstract	–
Panorama	4	13	Panorama	Filler	Abstract	–
Pech	1	11	Bad luck	Filler	Abstract	–
Planung	2	10	Planning	Filler	Abstract	–
Prozedur	3	14	Procedure	Filler	Abstract	–
Ruhe	2	9	Quiet	Filler	Abstract	–
Schicksal	2	10	Fate	Filler	Abstract	–
Sehnsucht	2	12	Nostalgia	Filler	Abstract	–
Sicherheit	3	8	Safety	Filler	Abstract	–
Vertrauen	3	9	Trust	Filler	Abstract	–
Zerstreuung	3	16	Distraction	filler	abstract	–
Zufall	2	11	Coincidence	Filler	Abstract	–
Zweifel	2	9	Doubt	Filler	Abstract	–
Kreuzung	2	11	Crossing	Filler	Abstract	–
Wohnwagen	3	13	Caravan	Filler	Abstract	–
Geländer	3	14	Railing	Filler	Concrete	–
Computer	3	9	Computer	Filler	Concrete	–
Postkarte	3	14	Postcard	Filler	Concrete	–
Lampe	2	13	Lamp	Filler	Concrete	–
Statue	2	13	Statue	Filler	Concrete	–
Theater	2	9	Theatre	Filler	Concrete	–
Nadel	2	13	Needle	Filler	Concrete	–
Moped	2	14	Moped	Filler	Concrete	–
Tür	1	9	Door	Filler	Concrete	–
Fernsehen	3	9	Watch TV	Filler	Concrete	–
Trinkglas	2	19	Drinking glass	Filler	Concrete	–
Hubschrauber	3	11	Helicopter	Filler	Concrete	–
Poster	2	13	Poster	Filler	Concrete	–
Masche	2	13	Mesh	Filler	Concrete	–
Geige	2	13	Violin	Filler	Concrete	–
Halstuch	2	16	Bandana	Filler	Concrete	–
Stecker	2	14	Plug	Filler	Concrete	–
Foto	2	8	Photo	Filler	Concrete	–
Geld	1	6	Money	Filler	Concrete	–
Produkt	2	10	Product	Filler	Concrete	–
Mantel	2	12	Coat	Filler	Concrete	–
Sack	1	12	Bag	Filler	Concrete	–
Zahnrad	2	17	Gear	Filler	Concrete	–
Kostüm	2	13	Costume	Filler	Concrete	–
Bleistift	2	14	Pencil	Filler	Concrete	–
Heft	1	12	Notebook	Filler	Concrete	–
Taschentuch	3	15	Handkerchief	Filler	Concrete	–
Zug	1	9	Train	Filler	Concrete	–
Buch	1	8	Book	Filler	Concrete	–
Kopfhörer	3	14	Headphone	Filler	Concrete	–

For each of the 64 non-filler words, a colour video was created using a Canon Legria HF S10 camcorder (Canon Inc., Tokyo, Japan). In each video, an actress performed a gesture that conveyed a word meaning. The actress was always positioned in the center of the video recording. She performed the gestures using head movements, movements of one or both arms or legs, fingers, or combinations of these body parts and maintained a neutral facial expression throughout each video. The word *bottle*, for example, was represented by the actress miming drinking from an imaginary bottle, and the word *triumph* was represented by the actress raising her right fist and inclining her body on the left. The actress began and ended each gesture by standing motionless with her arms at her sides and facing the video camera. Large gestures (e.g., steps or jumps) were restricted to a 1 m radius around the body’s starting position. Gestures used to convey the meanings of abstract words were agreed upon by three independent raters^99^. Videos lasted 4 s, but the actual execution of the gesture took about 1.5 to 2 s.

A black-and-white line drawing was also created for each of the 64 words. The line drawings conveyed word meanings by portraying humans, objects, or scenes. Pictures illustrating concrete nouns were mostly drawings of single objects, and pictures illustrating abstract nouns were often scenes. The complexity of the illustrations for concrete and abstract words was not matched, since similar differences are expected in natural encoding settings.

### Experimental design

The study utilized a 4 × 2 repeated measures design. Within-participant independent factors were encoding condition and word type. The encoding conditions were: Visual (V), Image (VI), Gesture Observation (VGO), Gesture Observation and self-Performance (VGOP). In the V condition, only the written word was presented. In the VI condition, written words were enriched by a black-and-white vignette depicting the correspondent object (for concrete words) or metaphorically representing the word meaning (for abstract words). In both the VGO and VGOP conditions, written words were accompanied by a video displaying an actress who performed an iconic (for concrete words) or metaphoric (for abstract words) gesture using both hands.

Four sets of stimuli for each type of words (concrete and abstract) were created, each including 8 words. The assignment of the words to the different sets was done such that words’ psycholinguistic parameters (i.e., number of syllables and familiarity scores) were homogeneous across experimental blocks. The order of encoding conditions was counterbalanced across participants.

### Procedure

The study consisted of an encoding phase followed by free recall and recognition memory tests. Participants then completed the same recognition task while EMG activity and eye movements were recorded.

#### Encoding phase

**Figure 5:**
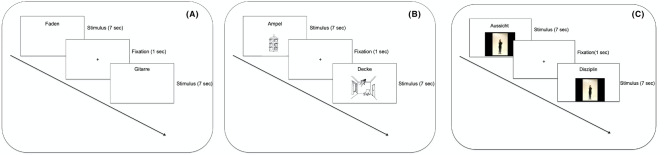
Word encoding procedure. (**A**) Visual condition—V; (**B**) Image condition—VI; (**C**) Gesture Observation—VGO, and Gesture Production conditions—VGOP. Instructions were: V condition—read aloud the word; VI condition—read the word and view the picture; VGO condition—read the word and watch the video; VGOP condition—stand up, read the word, watch at the video and perform the same gesture.

Participants sat in front of a 15″ laptop computer, at a distance of approximately 50 cm. In all of the conditions, participants viewed a German word written in black font against a white background (font: Arial, font size: 32 pt) that was presented at the top of the screen, horizontally aligned in the center. In the V condition, the written word was the only stimulus that was viewed by participants. In the VI condition, a picture was presented below the written word, in the middle of the screen. In both the VGO and VGOP conditions, a video of an actress performing a gesture was presented below the written word instead of a picture. The pictures and the videos appeared simultaneously with the written words. Stimuli were presented electronically using E-Prime 2.0 software (Psychology Software Tools, Pittsburgh, PA). Each stimulus (regardless of the encoding condition) lasted 7 s, and was followed by a fixation point (a cross displayed in the center of the screen) lasting 1 s. Instructions relevant for each condition were displayed before each encoding block: in the V condition, participants were instructed to read aloud the word; in the VI condition, they were asked to read the word and view the picture; in the VGO condition, participants were asked to read the word and watch the video; in the VGOP condition, participants were asked to stand up, read the word, watch at the video and perform the same gesture. Figure 5 depicts the four encoding conditions.

The training session comprised 8 blocks. For half of the participants, the first 4 blocks comprised concrete words and the last 4 blocks comprised abstract words, and vice versa for the other half of the participants. Trials were blocked by encoding condition, such that, within each set of 4 blocks, each block corresponded to one of the encoding conditions. Eight words were encoded in each block. Each stimulus in a given block was presented 7 times within the block, resulting in a total of 56 trials per block. The order of items within each block and the order of blocks within each session were randomized. The encoding phase lasted approximately 60 min.

#### Memory tests

Immediately after the encoding phase, participants completed a free recall and a recognition test. In the free recall test, participants were asked to write down all the words they remembered as they came to mind in any order. The amount of time given to complete the task was limited to 5 min. Next, the recognition test started. Words in the recognition test were presented using E-Prime software. The 64 target words and the 64 filler words were presented one at a time, in random order, in the center of the screen (black letters, font: Arial, font size: 32 pt; white background). Participants were asked to press, with either their right or left index finger, one of two target buttons (“p” and “q” on the keyboard) to indicate whether or not the displayed word was included in the set of trained words. The association of each button to “yes, it was included” and “no, it wasn’t included” responses was counterbalanced across participants. Response times were recorded.

#### EMG recognition task

After completing the free recall and recognition tests, participants completed another recognition task during which forearm EMG signals and eye movements were recorded. The forearm muscles were targeted with EMG for two main reasons. First, we were interested in the question of whether gesture performance during word encoding would increase motor resonance by comparison to other forms of encoding, and the forearms were seen as the most likely location to observe increases in EMG activity given the central role of the forearms in the performance of the gesture stimuli. Second, a pre-stimulus baseline period permitted task-related forearm EMG activity to be compared across conditions in relation to within-muscle control activity. Eye movements were recorded in order to allow the participants to complete the task by gazing in the direction of the correct answer on the screen, instead of using their forearm muscles to press buttons.

**Figure 6:**
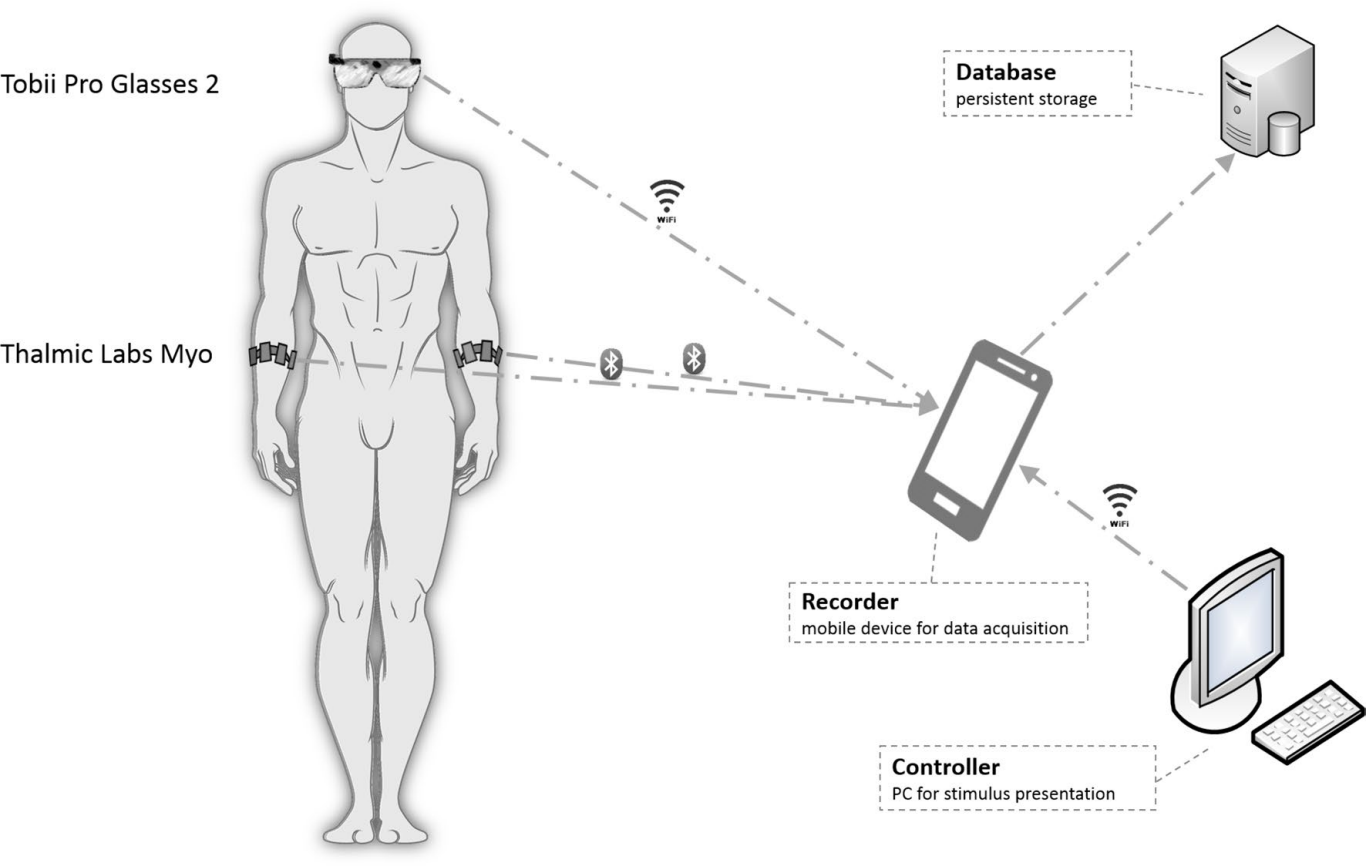
EMG recording and eye-tracking setup.

The EMG of the left and right forearm muscles was recorded using two Myo bracelets from Thalmic Labs (Kitchener, Canada), shown in Fig. 6. The wireless, flexible bracelets each contained 8 circular, equidistant aligned, medical grade stainless steel, single differential surface electrodes. These 8 electrodes deliver EMG data at a sampling rate of up to 200 Hz and a resolution of 8 bits signed.

Tobii Pro Glasses 2 from Tobii AB (Danderyd, Sweden) were used to record eye movements (Fig. 6). The Tobii system detects eye movements using four infrared cameras (two per eye) with a sampling rate of 50 Hz. The eye-tracking environment is recorded via an integrated full HD scene camera (1920 × 1080 pixels, 25 frames per s).

A desktop computer, with a 46-inch monitor referred to as the “Controller” (Fig. 6) was used to present the word stimuli. Stimulus presentation was implemented as a Java application. The Controller, along with the EMG bracelets and eye-tracking glasses, transmitted data to a mobile device referred to as the “Recorder”. The eye-tracking-glasses and the Controller used WiFi for data transmission, while the EMG bracelets used Bluetooth Low Energy. The Recorder time-logged all of the experimental events and uploaded data to a Database server. The complete setup is shown in Fig. 6.

Prior to starting the EMG recognition task, participants were outfitted with eye-tracking glasses and EMG bracelets. The EMG bracelets were aligned in position with the flexor digitorum superficialis muscle of each forearm. After calibration of the eye-tracking glasses, EMG reference data were acquired as the participants moved their arms or fingers into several positions. Participants were then asked to take a relaxed position in front of the screen, with their arms either relaxed on the table or, if preferred, on the chair’s armrests.

Each trial of the EMG recognition task began with a blank screen. After 0.9 s, a word appeared in the center of the screen (black letters, font: Arial, font size: 32 pt; white background). After another 2.05 s, boxes containing the words “Yes” and “No” appeared in the bottom left and right corners of the screen, 80 cm apart from each other. The word and the boxes remained on the screen for an additional 2.05 s before the next trial began. Participants were instructed to indicate whether they had learned that word during the training or not by focusing on the “Yes” or “No” boxes that appeared at the bottom of the screen on each trial. They were also asked to refrain from turning or tilting their head while completing the task. In order to familiarize the participants with the task, they completed 5 practice trials containing words that were not otherwise used in the study. During the task, the 64 target words and the 64 fillers were presented in a random order for each participant.

### Data analysis

All participants who completed the study and for whom eye-tracking measures were recorded for the entire experimental session (*n* = 28 participants) were included in the analyses.

We tested our hypotheses using linear mixed effects modelling. Linear mixed effects models were generated in R version 1.2.1335 using the ‘lme4’ package^101^. Generalized linear mixed effects models (function: glmer()) with a binomial error distribution were used to model binary dependent outcomes (i.e. recall accuracy). For each dependent variable, we performed backwards model selection to select the model’s random effects structure, beginning with random intercepts by subject and stimulus item and random slopes by subject and by stimulus item for the encoding condition factor and word type factor. We removed random effects terms that accounted for the least variance one by one until the fitted mixed model was no longer singular, i.e., until variances of one or more linear combinations of random effects were no longer (close to) zero. The final mixed effects model for all dependent measures included fixed effects of encoding condition and word type, as well as random intercepts by subject and stimulus item [*y* ~ encoding condition * word type + (1 | subject) + (1 | stimulus)].

Contrasts were coded using simple coding, i.e. ANOVA-style coding, such that the model coefficient represented the size of the contrast from a given predictor level to the (grand) mean (represented by the intercept). Following the procedure outlined by Alday et al.^102^, significance testing of effects was performed using Type-II Wald χ2 tests implemented in the ‘car’ package (function: Anova()^103^). Post-hoc Tukey tests were conducted using the ‘emmeans’ package^104^. The significance threshold was set to α = 0.05^105^.

#### Memory tests

For the free recall task, a score of 1 was assigned to words retrieved correctly, and a score of 0 was assigned to omitted words and intrusions, i.e. words that had not been included in the training. For the recognition task, response times were computed only for trials in which target words were correctly recognized as having been encoded during the training. Response times in the recognition task were computed as the time interval from word onset to button press. Trials were considered incorrect if the response was incorrect or if participants did not respond within 5 s of word presentation. Under this criterion, *M* = 6.7 trials per participant (*SD* = 5.2 trials) were identified as incorrect (*M* = 5.2%, *SD* = 4% of all trials).

#### EMG recognition task

To identify correct response trials, we computed mean x-coordinates of eye gaze locations within the time window that occurred 2.05 to 4.10 s post-word onset for each participant. Trials for which individual trial x-coordinate values exceeded 2 *SD* from each participant’s grand mean x-coordinate value were considered incorrect. Under this criterion, *M* = 3.5 target word trials (*SD* = 2.9 trials) per participant were identified as incorrect (*M* = 5.4%, *SD* = 4.6% of all target trials).

EMG activity during correct response trials was averaged across channels and segmented into epochs beginning 0.2 ms prior to word onsets and ending 2.05 s following word onsets. Activity was baselined within each epoch to EMG activity occurring in the 0.2 s prior to word onsets. Mean EMG activity during the period up to 2.05 s following words onsets was computed for each participant, encoding condition, and word type.

## Data Availability

The dataset generated and analysed during the current study are available from Dr. Mathias upon reasonable request.
